# Compassion Fatigue Among Nurses Working in Nursing Homes: A Descriptive Phenomenological Study

**DOI:** 10.1002/nop2.70330

**Published:** 2025-12-10

**Authors:** Nilay Ercan‐Şahin, Mücahide Öner

**Affiliations:** ^1^ Nursing Faculty Hacettepe University Ankara Turkey; ^2^ Nursing Department, Health Science Faculty Bitlis Eren University Bitlis Turkey

**Keywords:** compassion fatigue, descriptive phenomenological study, nurses, nursing home

## Abstract

**Aim:**

This study was conducted to explore the experiences of nurses in nursing homes regarding compassion fatigue.

**Method:**

A purposive sampling method was used to conduct semi‐structured in‐depth interviews with ten nurses working in a nursing home in Ankara, Turkiye between January and February 2024 for a descriptive phenomenological study. The collected data were analysed using thematic analysis.

**Results:**

Four themes emerged from thematic analysis: “Causes of compassion fatigue”, “Consequences of compassion fatigue”, “Burnout”, and **“**Cope with compassion fatigue”. The findings highlight that compassion fatigue has a lasting impact on nurses, affecting them both personally and professionally.

**Conclusion:**

It is important to develop programmes for the early detection of compassion fatigue among nurses working with older adults in nursing homes.

**Patient and Public Contributions:**

We thank all participants for their valuable input throughout the study.

## 1 Introduction

Nurses, comprising the largest and most vital group in healthcare, play a crucial role in meeting the complex needs of patients amid the growing demands of the healthcare system. However, they are susceptible to compassion fatigue, which involves difficulty managing emotional stress. Continued exposure to individuals experiencing suffering can result in emotional, physical, and mental exhaustion (Garcia 2022). Compassion fatigue occurs when individuals feel emotionally depleted and unable to give more of themselves. This statement implies a decrease in individual accomplishments that can lead to a pessimistic evaluation of one's work capacity (Rachel and Francesco 2018). Moreover, compassion is an ethical principle in nursing that leads to quality care, positive health outcomes, and patient satisfaction (Kirby et al. 2017). In addition to possessing practical and technical knowledge of nursing care, it is crucial for nurses to demonstrate compassion towards patients and their families. This compassionate approach is not only considered integral to professionalism in nursing but also essential in all healthcare settings where nurses practice (Bloomfield and Pegram 2015).

The literature includes numerous quantitative studies investigating compassion fatigue and its influencing factors among nurses working in various settings, such as oncology, emergency care, critical care, and pediatric nursing (Berger et al. 2015; Hunsaker et al. 2015; Sacco et al. 2015; Yu et al. 2016). Additionally, meta‐analyses on this subject have revealed that nurses frequently experience compassion fatigue (Algamdi 2022; Xie et al. 2021; Zhang et al. 2018).

Another challenging work environment for nurses, where they are at risk of experiencing compassion fatigue, is in nursing homes. In these settings, nurses provide care to older adults dealing with multiple emotional and physical losses, co‐morbidities, and end‐of‐life care needs (Raposo et al. 2017). Nurses caring for older adults may face challenges such as heavy workloads, staff shortages, limited resources, and high demands for quality care (Zimmerman et al. 2014). Moreover, compassion fatigue is triggered by unkind or prejudiced behaviour from patients or their families, patient suffering and death, and emotional conflicts arising from treatment (Fukumori et al. 2020). Upon reviewing the studies in this field, several quantitative investigations have assessed compassion fatigue among nurses working with older adults, revealing that it is a common issue among staff in this area (Kolthoff and Hickman 2017; Rachel and Francesco 2018; Sarabia‐Cobo et al. 2021). Additionally, Little's qualitative study involving 11 nurses and nurse aides working in long‐term care facilities demonstrated that these professionals are at risk for compassion fatigue (Little 2013). Similarly, another qualitative study of hospice and palliative care nurses indicated that these professionals experience compassion fatigue, emphasising the need for further research to understand the extent of the problem (Melvin 2012). However, no study has specifically focused on compassion fatigue among nursing home nurses in Turkey. Consequently, there is a significant gap in the literature regarding an in‐depth examination of the needs of nurses working in care homes in relation to compassion fatigue.

According to Figley's Compassion Fatigue Theory, empathic engagement—understanding and sharing the suffering of others—can lead to emotional exhaustion and compassion fatigue over (Figley 2013). Nurses working in nursing homes bear the responsibility of providing physical, emotional, and social care to older adults while witnessing their losses, chronic illnesses, and end‐of‐life (Fukumori et al. 2020; Raposo et al. 2017). This role necessitates a high level of empathic engagement, which increases their risk of developing compassion fatigue (Figley 2013). In Figley's model, empathy is viewed as both a fundamental cause of compassion fatigue and a critical component of caregiving. Understanding compassion fatigue among nurses working in nursing homes provides valuable insights into its causes, consequences, and coping strategies, making this theory a robust conceptual framework for this study (Figley 2013; Coetzee and Klopper 2010).

This study set out to examine the phenomenon of compassion fatigue among nurses employed in nursing home settings, with a specific emphasis on the research question: “How do nurses working in nursing homes experience compassion fatigue, and what are its implications at both personal and professional levels?” The insights derived from this exploration are expected to inform strategies aimed at enhancing the occupational well‐being of nurses and supporting their capacity to manage emotionally taxing experiences inherent in geriatric care. Furthermore, the study aims to contribute to the broader understanding of compassion fatigue within the nursing discipline and to guide the development of targeted interventions and resources. In this context, the identification of compassion fatigue is not merely viewed as a descriptive exercise, but rather as a critical step toward the formulation of evidence‐based responses—such as early intervention programs, institutional support mechanisms, and self‐care‐oriented educational initiatives—through the delineation of the organisational and emotional determinants underlying this phenomenon.

## 2 Methods

### 2.1 Study Design

In this study, a descriptive phenomenological approach was used to explore the experiences of nurses in nursing homes regarding compassion fatigue. Researchers chose this design for the study as it allows exploration of people's experiences and how they make sense of them (Creswell and Poth 2016). The study was reported according to the Consolidated Criteria for Reporting Qualitative Research (COREQ) Checklist (Booth et al. 2014). Adherence to COREQ ensured that all stages—from participant selection to data analysis—were presented in a clear and replicable way, thereby enhancing the credibility of the study and enabling the applicability of the findings to similar settings.

### 2.2 Settings

The study was conducted at a nursing home in Ankara, Turkiye, between January and February 2024. As one of the largest facilities of its kind in Ankara, it provides care to a significant number of nursing home residents. The institution houses 319 older adult residents, approximately 200 of whom are bedridden.

### 2.3 Participants

This study was conducted with 16 nurses employed at a nursing home, where they work 12hours day and evening shifts. Each nurse is responsible for the care of 30 to 40 residents. The sample was selected using purposive sampling, with a focus on qualitative data collection. All 16 nurses were invited to participate in the study. Face‐to‐face communication was established with nurses working at the nursing home, and they were provided with information about the study. Interviews were conducted with those who agreed to participate. The inclusion criteria for the sample were as follows: at least one year of work experience in a nursing home setting and a willingness to voluntarily participate in the study.

### 2.4 Data Collection

**FIGURE 1:**
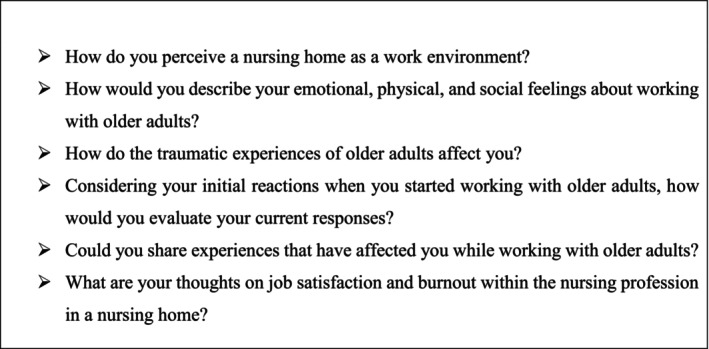
Contents of semi‐structured interview guide.

Data were collected through open‐ended semi‐structured interviews following an interview guide (Figure 1). Before each interview, participants provided consent and completed a demographic information form indicating their age, gender, educational level, years of nursing experience, and years of nursing experience in nursing homes. Using the semi‐structured interview guide, each participant then took part in a one‐time, in‐depth, face‐to‐face interview. The principal investigator, a female with a PhD degree working as an associate professor in the public health nursing department at a university, conducted the interviews in a quite private room at the nursing home to ensure participant privacy. Field notes were taken during or after the interviews. Each interview lasted approximately 35–40 min and was audio‐recorded. In qualitative research, interview durations are typically recommended to be between 30 and 90 min. This duration allows for a detailed exploration of the participant's experiences while maintaining engagement and focus throughout the interview (Creswell and Poth 2016). To explore nurses' views and experiences regarding compassion fatigue, the concept of “compassion fatigue” was not directly included in the questions. Instead, the focus was on their experiences while providing care to older adults, how these experiences have changed over time, how they affect them physically, mentally, and socially, and their feelings about the challenges they face while caring for older adults. Additionally, during the interviews, participants were asked to elaborate on points that were not clearly expressed, with the help of questions such as “What do you mean when you say […]?” and “Could you tell me a little bit more about that?”

According to Creswell, phenomenological studies should include individuals who have thoroughly experienced the phenomenon, and the recommended number of participants can range from 5 to 25 (Creswell and Poth 2016). However, in this study, in‐depth interviews continued until data saturation was reached, which was defined as the point when sufficient information was obtained to address the purpose of the study, and the collection of new data and further coding were no longer feasible (Guest et al. 2006). After the tenth interview, the researchers noticed no new data regarding repetitive elements and experiences of compassion fatigue in the participants' responses, leading to the termination of the data collection process. Consequently, the interviews were terminated after the 10th interview. The transcripts were returned to the nurses for their review. Table 1 describes the main sociodemographic and occupational characteristics of the participants.

**TABLE 1:** Characteristics of participants.

Participants	Age	Gender	Educational level	Years of nursing experience	Years of nursing experience in nursing home
1	28	Female	Undergraduate	3	1.5
2	30	Female	High school	11	9
3	30	Female	High school	12	8
4	25	Female	High school	8	1.5
5	24	Male	High school	5	3
6	32	Female	Master	2	2
7	29	Male	High school	9	5
8	25	Female	Undergraduate	2	2
9	34	Female	High school	14	11
10	36	Female	Undergraduate	13	3

### 2.5 Data Analysis

The audio‐recorded semi‐structured in‐depth individual interviews were transcribed verbatim by the first author, and the second author cross‐checked the transcripts. The data analysis software MAXQDA was used to organise the interviews and to code the data. Braun and Clarke's (2006) thematic analysis was used to analyse the data. Thematic analysis was chosen for its effectiveness in unveiling patterns within data sets that are significant to the phenomenon in this descriptive qualitative study (Braun and Clarke 2006). Firstly, the authors read the entire transcripts. Secondly, the sentences related to the phenomenon under inquiry were highlighted.

Thirdly, the authors read each line of the text and interpreted it to decipher what it revealed about the phenomenon. Subsequently, codes were generated and defined based on the aim of the study, leading to the derivation of themes from the data. Two independent authors generated and checked the codes until they reached a consensus. Then, the codes were grouped into themes and sub‐themes. Finally, four main themes and sub‐themes that reflected the content of the interviews were developed. Written notes and observations during data collection were compared with the data from group discussions, and the findings of this study were corroborated.

### 2.6 Trustworthiness

To demonstrate reliability, we opted to use the original criteria presented by Lincoln and Guba, which are widely accepted and easily recognisable (Guba and Lincoln 1982). Lincoln and Guba emphasised that a study's reliability can be ensured by considering factors such as credibility, transferability, consistency, and verifiability. In this context, it is aimed to ensure credibility by providing a long‐term interaction. To improve the transferability, purposive sampling methods were used along with a detailed description. The study provides detailed information on the research stages, materials, and questions to enable repetition. Thus, the principle of transferability was met by auditing the study. To ensure verifiability, all data collection tools, transcripts, and notes used in the study were retained for further review when necessary.

### 2.7 Ethical Considerations

Approval from the ethics committee of the university (REDACTED, dated: July 03, 2023) was obtained, and written permission was also obtained from the nursing home and rehabilitation centre where the study was conducted. Verbal and written informed consent were obtained from all participants. All the written and audio recordings were securely stored as copies.

## 3 Results

**FIGURE 2:**
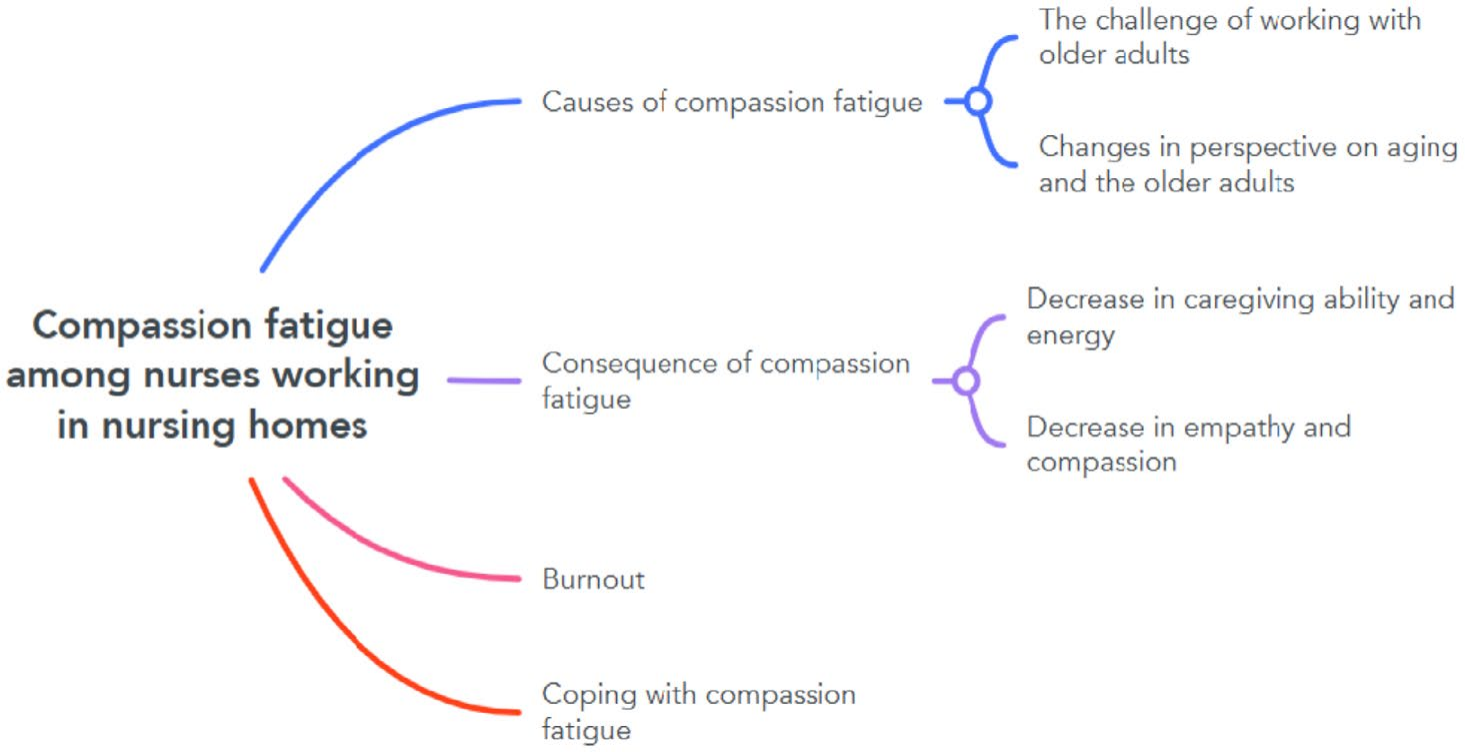
Themes and sub‐themes of compassion fatigue among nurses working in nursing homes.

Four themes and five sub‐themes regarding compassion fatigue among nurses working in nursing homes have emerged and are presented in Figure 2.

### 3.1 Theme 1. Causes of Compassion Fatigue

According to feedback from nurses employed in nursing homes, the challenges of working with the older adult, changes in perspective on ageing and the older adult, contribute to compassion fatigue among them.

#### 3.1.1 Subtheme 1. The Challenge of Working With Older Adults

All nurses highlighted the significant challenges they face when caring for older adults, particularly in the nursing home setting. They noted that residing in a nursing home often amplifies the difficulties associated with caregiving, making it more demanding than in other environments. The ageing process of the residents, accompanied by physical and cognitive declines, further intensifies these challenges, requiring more intensive care and emotional investment. Consequently, the nurses expressed that the emotional and physical strain of caring for older adults in such an environment frequently leads to profound disappointment. They shared that the complexities of providing care, along with the challenges of managing residents' conditions, can diminish their sense of fulfilment and satisfaction in their work.

… Immmm, as you know, working with people is challenging. Working with sick people is even more challenging. Working with sick older people is much, much more challenging (P5)

Working with older adults… (pausing to reflect) …hmm… I'm not entirely certain… I mean, there aren't many positive aspects of working with older adults. … (Continues to contemplate)… (P7)

Some nurses mentioned empathising with the older adult, speculating that they might face similar situations in the future, and this realisation sparked concerns about their own ageing process.

Sometimes when I engage in intense empathy, I feel like I'm drowning in it. I mean, I worry that I might end up like that too… (P8)

Some nurses elaborated on how certain traits specific to the nursing home residents made it challenging for them to perform their duties effectively, prompting them to either step back.

The stubbornness of the older adult, can indeed be very draining. It truly tests your patience, leaving you with little tolerance for people or anything else. When working with the older adults you often find yourself holding back. (P2)

Sometimes the residents say to me, “You're working here with my money.” I'm not working here with your money. We're actually working to eliminate your sense of abandonment. You are dependent on me, and I'm doing more than a thousand times my best to help you without making you feel it, yet in return, I am thinking that I face ingratitude from the older adults. (P10)

#### 3.1.2 Subtheme 2. Changes in Perspective on Ageing and the Older Adults

Nurses have expressed the numerous challenges they face when working with older adults, pointing out that their perceptions towards ageing and the older adult have undergone significant shifts over time. The unchangeable nature of the conditions affecting older adults further exacerbates the emotional toll on the nurses, contributing to the escalation of compassion fatigue. The inherent challenges of caring for individuals whose conditions are often irreversible or deteriorating make it increasingly difficult for nurses to maintain emotional resilience, ultimately leading to heightened feelings of exhaustion and emotional strain.

………So, when I first began to work here, I was 23 years old. I used to look at the older adult with, so to speak, the perspective of affectionate uncles and aunts, but to be honest, indeed we make the individual innocent in our own eyes just because they are old. Just because someone is old doesn't mean they're very innocent or very well‐intentioned. They don't always think good thoughts, they don't refrain from swearing, you know, things like that. I no longer make people innocent just because they're old…… (P5)

…I'm not as optimistic towards the older adults as I used to be. For example, you give your seat to the older adults on the bus, right? I don't want to give it away right now… (P5)

………I currently perceive aging as a sickness, I believe it renders people very vulnerable… (P6)

### 3.2 Theme 2. Consequences of Compassion Fatigue

Nurses working with older adults in nursing homes reported that, over time, they began to feel a decline in their ability to provide high‐quality care. Many of them expressed experiencing a profound sense of exhaustion and depletion, both physically and emotionally, leading them to feel as if they were running out of energy. Furthermore, they noted a noticeable decrease in their levels of empathy and compassion towards the residents. This gradual erosion of their emotional resources was seen as a significant challenge in their professional lives, affecting their overall well‐being and the quality of care they were able to provide.

#### 3.2.1 Subtheme 2.1. Decrease in Caregiving Ability and Energy

Almost all nurses indicated that, due to the constant repetition of requests from the older adult, they reach a point where they can no longer attend to them, leading to a depletion of their energy.

it's exhausting for me when the residents keep repeating the same things, the same troubles, not understanding me. Dealing with the same questions, the same situations drains my energy (P7)

Here, it feels like they constantly need to tell us about a problem, like ‘my stomach hurts, my’ back hurts, can you look at my back? Even though there might not actually be any pain, they behave as if there could be, so, I feel like my energy is drained (P6)

Sometimes I feel really overwhelmed. Actually, it's not that I don't like it, I love working here. But sometimes, you know, things pile up, and there are times when I just don't want to see the older adult anymore, I don't want to talk anymore, I feel like I've run out of strength (P2)

#### 3.2.2 Subtheme 2.2. Decrease in Empathy and Compassion

The nurses expressed that their interactions with the older adult changed over time, finding it challenging to maintain the same level of friendliness or tolerance as they did initially. Additionally, they mentioned a tendency to normalise older adults' experiences or actions.

After a while, you don't think too much. At the beginning, you think a lot, you get upset, and so on, but after a while, everything kind of normalizes. You see only it as task. (P3)

I used to be much more cheerful or smiling, trying to handle things with a smile while working. Now, of course, I'm not behaving badly, never. But what I used to try to handle with a smile or a cheerful face, I now try to handle a bit more quietly. So, if I say I've withdrawn myself a notch more, it wouldn't be a lie. (P10)

At first, the death of a resident, which used to deeply sadden me, has become much more normalized. Or when an older adult person keeps repeating the same thing to us, we no longer pay attention. Because we're constantly exposed to the same thing, the same words, it becomes trivial (P1)

At first, we could tolerate everything about them. Now, when something is added on top of that, the tolerance level decreases. It's as if compassion is diminishing. It really happens with a few older adults here (P2)

### 3.3 Theme 3. Burnout

The nurses shared their growing reluctance to continue working in the nursing home for extended periods due to the various challenges they faced in caring for older adults. They described how these challenges, such as dealing with complex medical conditions, emotional demands, and the physical toll of their work, gradually took a significant psychological and emotional toll on them. As a result, many of them experienced increasing levels of psychological exhaustion and burnout, leading to a diminished sense of fulfilment and a desire to distance themselves from their duties.

For me, this isn't a place where I can work for many years. It's a psychologically exhausting place that seriously scares me for the future (P9)

I am also experiencing burnout from working here. It's very depressing. I am in very different psychology among very different people, I have become very stagnant. I turned into a completely different person (P9)

As a nurse working in a nursing home, I definitely do not want to work for many years. I have been working for two years, but I already feel burnout (P1)

I am psychologically exhausted because I have to be patient and tolerate every time (P8)

### 3.4 Theme 4. Coping With Compassion Fatigue

Despite experiencing burnout or compassion fatigue from working with the nursing home residents, nurses acknowledged and accepted this reality. Some likened it to the dynamics of a mother–child relationship, while others attributed it to the natural progression of ageing. Additionally, some nurses attempted to cope with the situation by emphasising the importance of respecting the older adults due to their age.

We always try to approach things positively. I try to understand them most of the time. Sometimes they can be difficult. They shout, they curse, but we always have to endure it, both because of their age and the nature of aging (P2)

They are with us until they pass away. They need us. They are dependent on us, and we can think of it as a relationship between a child and a mother at home. There's unlimited favoritism. It's the same here (P2)

Sometimes I do get annoyed involuntarily, but I end up being the one who tolerates it. There's nothing I can do; they're older adult, and we're here for them. They are our older adult's, and ultimately, respect is necessary (P4)

## 4 Discussion

Nursing home nurses care for older adults who are dealing with various emotional and physical losses, co‐morbidities, and end‐of‐life care needs, all of which can increase the risk of compassion fatigue among nurses (Cagle et al. 2017; Raposo et al. 2017). The objective of this study was to explore the experience of compassion fatigue among nursing home nurses, examining not only how it affects them but also the various factors that contribute to its development. The findings revealed that compassion fatigue has a profound and lasting impact on nurses, influencing both their personal well‐being and professional effectiveness. As a result of the analysis, four key themes emerged, shedding light on the complex nature of this phenomenon and providing a deeper understanding of how it affects nurses' emotional, psychological, and social experiences in their day‐to‐day work.

One of the most significant findings of this study is that nurses underscored the numerous challenges they face in caring for nursing home residents. They shared that these challenges, combined with the emotional demands of their work, had a profound impact on their views toward both ageing and older adults. Initially, many of the nurses had a compassionate and understanding perspective toward older adults, both within the institution and in society. However, over time, their experiences in the nursing home environment led to a shift in their attitudes, causing them to create emotional distance from the residents they cared for. This emotional detachment, while perhaps a coping mechanism, resulted in negative feelings not only toward the older adult residents within the institution but also toward older adults in the broader society. The nurses reported that the emotional strain of their work, compounded by the continuous exposure to suffering and end‐of‐life care, made it difficult to maintain the same level of empathy and connection they had previously felt. In line with similar studies, it is suggested that nurses may adopt tougher behavioural approaches as a protective mechanism against emotionally taxing situations (Duarte and Pinto‐Gouveia 2017; Gustafsson and Hemberg 2022).

Compassion fatigue is recognised as holistic exhaustion, characterised by a physical decline in energy and endurance, an emotional decline in empathetic ability and emotional exhaustion, and a spiritual decline as one feels hopeless or helpless to recover, which results from chronic exposure to others' suffering (Peters 2018). Compassion fatigue can diminish major attributes of effective nursing—empathy and caring—which are essential for building trust in the nurse/patient relationship (Salmond et al. 2019). Similarly, in this study, nurses working with older adults reported that as a direct consequence of experiencing compassion fatigue, both their ability to provide effective care and their overall energy levels were significantly diminished. Additionally, they experienced a decline in their capacity for empathy and compassion, which are essential components of quality care. These negative effects not only impacted their professional performance but also led to a sense of emotional exhaustion, making it increasingly difficult for them to maintain the same level of care and emotional connection with the older adult individuals they serve. In the context of this study, nurses reported being responsible for 30 to 40 older adults during a single shift. This high patient‐to‐nurse ratio makes it extremely challenging to attend to each individual's needs, actively listen to them, and provide holistic care. Under such demanding workloads, allocating time for anything beyond essential medical treatments is often unrealistic, which further hinders the provision of compassionate and person‐centred care.

Another significant finding of this study is that nurses experienced burnout, a condition that is characterised by negative attitudes and behaviours that significantly hinder job performance in nursing, often as a direct result of work‐related stress (Khamisa et al. 2016). Burnout is particularly prevalent among healthcare professionals who are responsible for caring for patients with complex needs, such as older adults with dementia, who require intensive attention and care. These professionals frequently experience a decrease in compassion satisfaction, an increase in compassion fatigue, and a higher likelihood of burnout (Hamama et al. 2019). Moreover, it has been noted that the prevalence of burnout and compassion fatigue is notably higher in nursing homes compared to other care settings, indicating the particularly demanding nature of this work environment (Sarabia‐Cobo et al. 2021; White et al. 2020). In line with these findings, this study revealed that nurses in the nursing home setting expressed a reluctance to remain in the institution for extended periods. They reported struggling to maintain patience, often feeling overwhelmed, and ultimately experiencing burnout during their tenure. This highlights the critical need for intervention strategies to address burnout and support nursing staff in managing the emotional demands of their roles.

The final significant finding of this study highlights that nurses have developed a range of coping strategies to manage the compassion fatigue they experience, allowing them to maintain a high standard of care for nursing home residents despite the emotional toll. Research has shown that compassion fatigue among nurses can lead to several detrimental outcomes, including poor decision‐making, a loss of empathy, decreased productivity, and a significant reduction in the quality of care provided (Jenkins and Warren 2012; Kelly et al. 2015; Shin et al. 2014). To counteract these negative effects, many nurses reported that one of the most effective coping strategies involved accepting the older adult as they are and showing them respect. This approach helps nurses maintain their emotional well‐being and prevents the onset of burnout. By adopting these coping mechanisms, nurses are able to continue offering quality care while protecting themselves from the emotional exhaustion that can result from the demanding nature of their work. Ultimately, these strategies help nurses motivate themselves to remain in their roles and continue providing care in the nursing home setting.

### 4.1 Limitations

There are a few limitations that should be considered when interpreting the results. First, this study's generalisability is limited by its qualitative nature, as it primarily reflects the perceptions and ideas of nursing home nurses. Second, the limitation is that the informants were mostly female nurses, but since the gender perspective was not a focus, there is no information on this issue. Third, the nurses were recruited from a single nursing home. However, the sample is considered adequate because each nurse's experience of compassion fatigue while caring for older adults in a nursing home is unique and cannot be replicated.

## 5 Conclusion

It has been revealed that compassion fatigue often leads to negative outcomes such as anger, withdrawal, and burnout among nursing home nurses. Despite these challenges, nurses strive to ensure that the quality of care they provide to the nursing home residents is not negatively affected. However, working with the older adults in a nursing home environment is psychologically exhausting for them.

These findings have significant implications for practice. It is important to develop programmes for the early detection of compassion fatigue among nurses working with older adults in nursing homes, where constant suffering is a characteristic feature. These programmes not only encourage the normalisation of sharing difficult emotions among professionals but also have the potential to prevent the consequences of compassion fatigue, such as burnout, frustration, and the desire to leave the profession in the future. Moreover, the findings suggest the need for a deliberate effort to cultivate a supportive environment that mitigates professional burnout and compassion fatigue while fostering compassion satisfaction. Policies and procedures should prioritise supporting nursing home nurses in managing both work and personal responsibilities, ensuring adequate time off, offering assignment rotations, flexible scheduling, educational benefits, and other opportunities for self‐care and professional development.

## Author Contributions

Study design: Nilay Ercan‐Şahin and Mücahide Öner. Data collection: Nilay Ercan Şahin. Data analysis: Nilay Ercan Şahin and Mücahide Öner. Study supervision: Nilay Ercan Şahin. Manuscript writing: Nilay Ercan Şahin and Mücahide Öner. Critical revisions for important intellectual content: Nilay Ercan Şahin and Mücahide Öner.

## Ethics Statement

Approval from the ethics committee at the university (number: E.3852, dated: July 03, 2023) was obtained, and written permission was also obtained from the nursing home and rehabilitation centre where the study was conducted. Verbal and written informed consent were obtained from all participants. All the written and audio recordings were securely stored as copies.

## Conflicts of Interest

The authors declare no conflicts of interest.

## Data Availability

The data that support the findings of this study are available from the corresponding author upon reasonable request.
